# Impact of a standardized protocol for the Management of Prolonged Neonatal Jaundice in a regional setting: an interventional quasi-experimental study

**DOI:** 10.1186/s12887-019-1550-3

**Published:** 2019-05-29

**Authors:** Hui-Siu Tan, Inthira-Sankari Balasubramaniam, Amar-Singh HSS, May-Luu Yeong, Chii-Chii Chew, Ranjit-Kaur Praim Singh, Ai-Yuin Leow, Fatimahtuz-Zahrah Muhamad Damanhuri, Santhi Verasingam

**Affiliations:** 1 Paediatric Department, Hospital Teluk Intan, Teluk Intan, Perak Malaysia; 2 Paediatric Department, Hospital Slim River, Slim River, Perak Malaysia; 3 Paediatric Department, Hospital Raja Permaisuri Bainun, Ipoh, Perak Malaysia; 4 Clinical Research Centre, Ipoh, Perak Malaysia; 5 Public Health Department, Medicine Faculty, Universiti Kebangsaan, Kuala Lumpur, Malaysia; 6 Perak State Health Department, Ipoh, Malaysia

## Abstract

**Background:**

Prolonged neonatal jaundice (PNNJ) is often caused by breast milk jaundice, but it could also point to other serious conditions (biliary atresia, congenital hypothyroidism). When babies with PNNJ receive a routine set of laboratory investigations to detect serious but uncommon conditions, there is always a tendency to over-investigate a large number of well, breastfed babies. A local unpublished survey in Perak state of Malaysia revealed that the diagnostic criteria and initial management of PNNJ were not standardized. This study aims to evaluate and improve the current management of PNNJ in the administrative region of Perak.

**Methods:**

A 3-phase quasi-experimental community study was conducted from April 2012 to June 2013. Phase l was a cross-sectional study to review the current practice of PNNJ management. Phase ll was an interventional phase involving the implementation of a new protocol. Phase lll was a 6 months post-interventional audit. A registry of PNNJ was implemented to record the incidence rate. A self-reporting surveillance system was put in place to receive any reports of biliary atresia, urinary tract infection, or congenital hypothyroidism cases.

**Results:**

In Phase I, 12 hospitals responded, and 199 case notes were reviewed. In Phase II, a new protocol was developed and implemented in all government health facilities in Perak. In Phase III, the 6-month post-intervention audit showed that there were significant improvements when comparing mean scores of pre- and post-intervention: history taking scores (*p* < 0.001), family history details (*p* < 0.05), physical examination documentation (*p* < 0.001), and total investigations done per patient (from 9.01 to 5.81, *p* < 0.001). The total number of patient visits reduced from 2.46 to 2.2 per patient. The incidence of PNNJ was found to be high (incidence rate of 158 per 1000 live births).

**Conclusions:**

The new protocol standardized and improved the quality of care with better clinical assessment and a reduction in unnecessary laboratory investigations.

**Trial registration:**

Research registration number: NMRR-12-105-11288.

**Electronic supplementary material:**

The online version of this article (10.1186/s12887-019-1550-3) contains supplementary material, which is available to authorized users.

## Background

Prolonged neonatal jaundice (PNNJ) is defined as visible jaundice with yellowish staining of the skin, mucous membrane and conjunctival icterus or serum bilirubin > 85 μmol/L that persists beyond 14 days of life in a term baby and 21 days in a preterm baby [1, 2].

Breast milk jaundice is the most common cause of PNNJ. It is almost always benign [3], presenting as prolonged unconjugated hyperbilirubinaemia, and occurs in up to one-third of healthy breastfed newborns [4]. It develops as the result of poor calories intake associated with breast-feeding difficulties [5], liver immaturity and the inhibitory effect of mother’s milk in the clearance of unconjugated bilirubin [6].

Prolonged neonatal jaundice could also be an early presentation of serious conditions such as biliary atresia [7, 8]. The incidence of biliary atresia varies worldwide from 1 in 6000 live births in Taiwan, 1 in 12,000 in the United States, 1 in 17,000 in the United Kingdom [9], 1 in 18,000 in Europe and 1 in 19,000 in Canada [10]. It can rapidly lead to liver cirrhosis and liver failure if left untreated. However, outcomes of babies with biliary atresia benefit from early diagnosis and hence babies with prolonged neonatal jaundice, and specifically babies with prolonged conjugated hyperbilirubinaemia need to be investigated to rule out biliary atresia [11–15]. Other pathological causes of prolonged neonatal jaundice are urinary tract infection, sepsis, congenital hypothyroidism, metabolic and haemolytic disorders [16].

A local unpublished survey among medical officers and staff nurses in Perak region had shown that the knowledge of health care providers in managing babies with PNNJ was not satisfactory. Majority of the personnel from health clinics (71%) and hospital (83%) were unable to give accurate diagnostic criteria. The study also showed that the management of PNNJ was not standardised. Seventy percent of health staff would refer all babies straight to specialist hospitals without conducting a preliminary investigation while the remaining 30% would be conducting some investigations before referring. Over investigation among hospital staff occurred in 50%, with medical officers ordering 8 or more laboratory investigation in the initial workup [17].

The current Malaysian and many international guidelines (Additional file 2 Table S1) state that babies with PNNJ should receive a routine set of laboratory investigations to detect serious conditions such as biliary atresia, without much emphasis on clinical assessment. A large number of well breastfed babies will similarly undergo these tests. Of note, these tests include urine culture which sometimes leads on to unnecessary treatment because of the false positive result [18].

On the other hand, despite this standard list of laboratory investigations there were still missed cases and cases with late diagnosis of biliary atresia [19, 20].

So how do we balance the need to detect serious conditions but not to over-investigate well babies? At present, the only effective method for early detection of biliary atresia is the universal stool colour screening and registry, which has been implemented in Taiwan in recent years [21]. In Bath (UK), the Paediatric Team in Royal United Hospital has adopted an approach where only babies with suspected abnormal clinical findings and history would be investigated further. Well, term babies will only require a simple laboratory investigation, which is total serum bilirubin with differential (direct and indirect levels) [22].

This successful strategy of using clinical assessment of risk factors in the evaluation and management of PNNJ has encouraged us to revise our pre-existing practices and develop a new protocol that standardises the management of prolonged neonatal jaundice.

## Methods

### Study design and setting

**Fig. 1:**
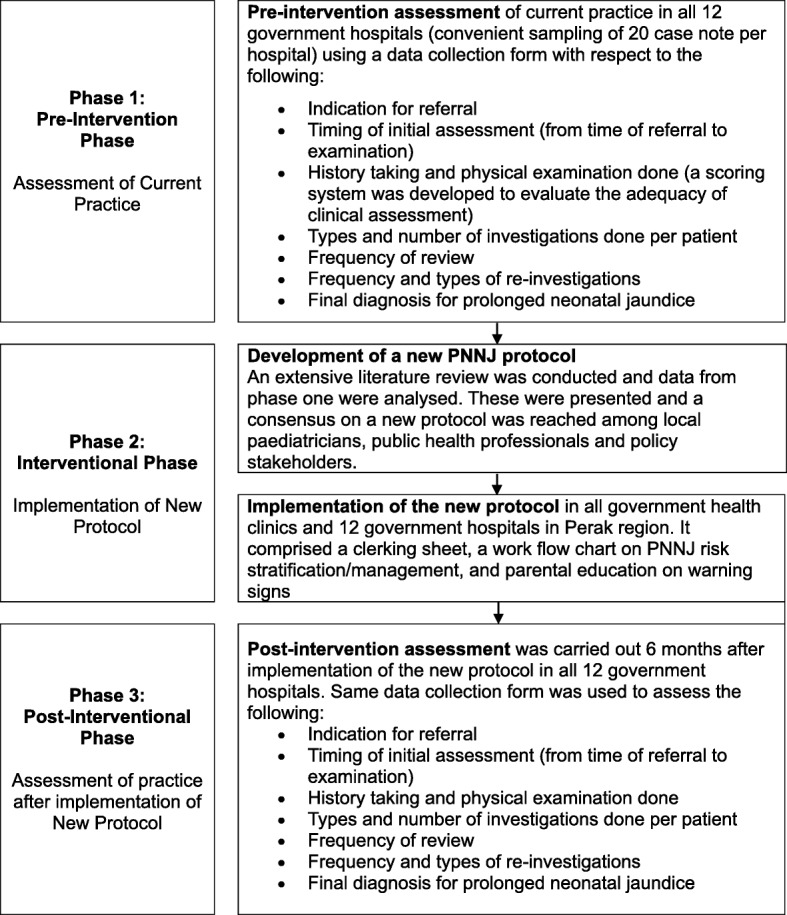
Methodology flow chart. The flow diagram illustrated the method of conducting this study in 3 different phases

This was a community quasi-experimental study conducted in 3 phases from April 2012 to June 2013, aimed at evaluating and improving the management of PNNJ in the Perak region, Malaysia. A flow chart of the methodology is illustrated in Fig. 1.

In Phase I, the pre-interventional phase, the current practices of PNNJ management at the selected sites were assessed. For each site, 20 most recent PNNJ case notes were reviewed using a pre-tested data collection form (Additional file 1). This form evaluated how babies with PNNJ were diagnosed, assessed and managed. The form was filled in by paediatricians or by a medical officer under the supervision of visiting paediatricians if in non-specialist hospitals.

Evaluation of patient history taking, family history taking and physical examinations (Additional file 1) obtained from the case notes were summed up in the form of a score. This score was based on important points that were relevant to the assessment of PNNJ. The maximum score was 5 for history taking, 4 for family history taking, and 5 for physical examination. The higher the score, the more complete was the clinical assessment.

**Fig. 2:**
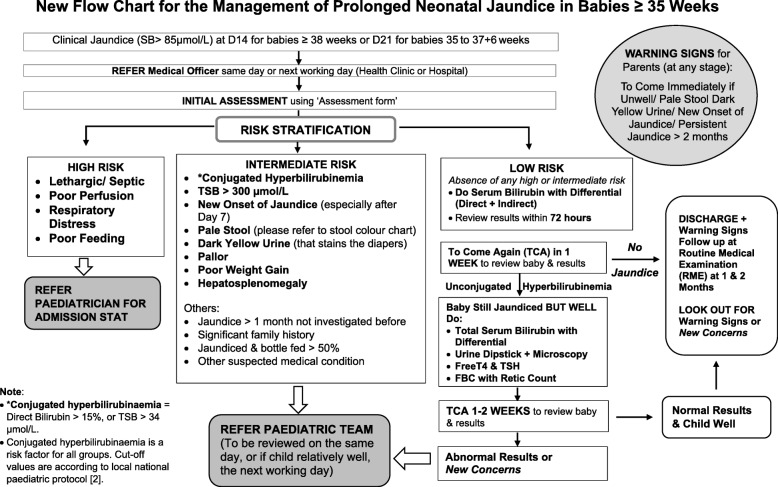
New protocol flow chart for the management of PNNJ. New protocol consists of a flow chart and an assessment form

**Fig. 3:**
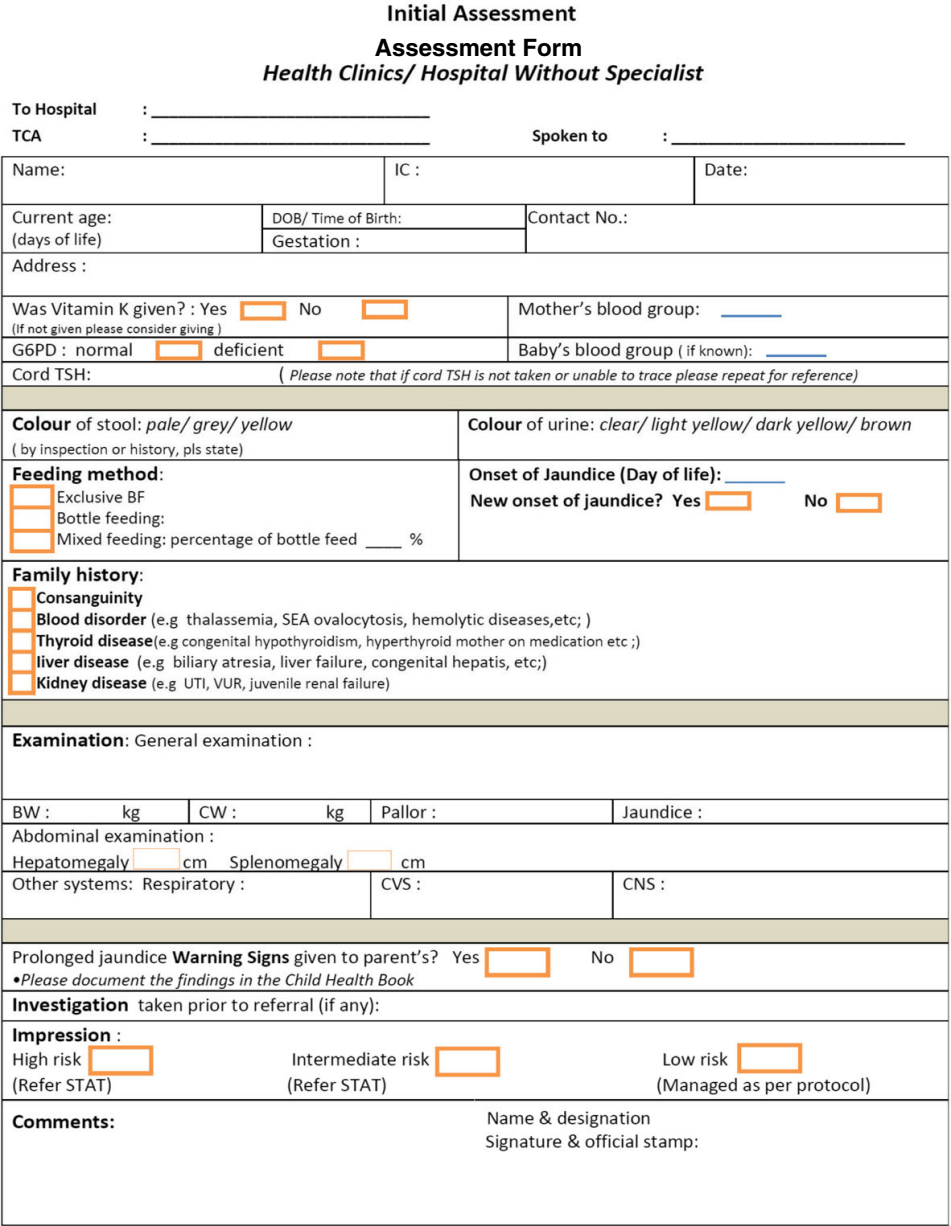
New protocol assessment form for the management of PNNJ in health clinics and hospitals without specialists

In Phase II, the interventional phase, a new protocol was developed based on analysis of data from Phase I, extensive literature review (see Table 1 and Additional file 2 Table S1, Additional file 3 Table S2, Additional file 4 Table S3, Additional file 5 Table S4 and Additional file 6 Table S5), and consensus from the stakeholders (local paediatricians, public health professionals, and local policy stakeholders). This new protocol focused on the management of PNNJ according to risk stratification and on educating parents about warning signs (unwell baby/ pale stool dark yellow urine/ new onset of jaundice/ persistent jaundice > 2 months). It comprised a flow chart for PNNJ management (Fig. 2) and an assessment form (Fig. 3), which were distributed together and was implemented in all 322 health clinics and 12 hospitals in the Perak region on September 2012.

**Table 1:** Differences between the current practice and the new protocol for PNNJ

No	Issue	Current Practice	New Protocol	Rationale for Change
1.	What clinical assessment and laboratory investigations are needed in the initial assessment of PNNJ?	Clinical assessment is not emphasised, and a routine list of laboratory investigation is done according to local/national protocol for all term babies with jaundice at 14 days of life.	Low risk babies At day 14*:* Do a complete clinical assessment using the assessment form and take total serum bilirubin with differential At day 21 if still jaundice: Repeat clinical assessment and carry out a simple list of lab investigation - Total serum bilirubin with differentials - Full blood count and reticulocyte count - Urine dipstick & microscopy test and - Free T4, TSH Intermediate/ high risk babies Refer to Paediatric team for further management	New system aims to focus on good clinical assessment. In well, breastfed term babies half of them will have jaundice resolved by 21 days of life [30]. Prompt referral of babies with risks and unwell babies to paediatricians.
2.	Is there a checklist for clinical assessment?	No	Yes, serves both as a checking list and referral sheet.	Ensure all essential clinical assessments are done for risk stratification
3.	Where could the initial assessment take place?	Paediatric clinics only.	Any nearby health clinics or district hospitals.	This aims to empower health clinics/ district hospitals to do the initial clinical assessment and workup and follow up on the low-risk babies. Specialist clinics will focus more on intermediate or high-risk cases.
4.	Heel prick capillary bilirubin vs total serum bilirubin with differential	Babies with PNNJ undergo repeated heel-prick capillary bilirubin in the health clinics, until the jaundice resolved.	Total serum bilirubin with differential is needed at 14 days and only repeated as necessary	Main aim of total serum bilirubin with differential is to pick up conjugated hyperbilirubinaemia [2] Heel-prick capillary bilirubin is not useful in the management of PNNJ.
5.	Urine sampling	Babies with PNNJ undergo urine culture, whereby sampling is done by clean catch, bladder catheterization or suprapubic aspiration.	Only urine dipstick & microscopy test and is needed. Sampling via urine bag is acceptable. Urine culture will be considered for suspected cases [18].	The incidence of UTI in asymptomatic, afebrile and jaundiced babies ranged from 5.5–21% [31]. There is a role of urine dipstick & microscopy only in the screening of UTI in well, jaundiced babies [18].
6.	Thyroid function tests (Free T4/ TSH)	This is conducted for all babies with PNNJ at day 14	This is conducted for all intermediate or high-risk babies and low risk babies if still jaundice at day 21	Thyroid function test is necessary to detect congenital hypothyroidism cases that are missed by the newborn screening programme [32].
7.	Full blood picture	This is conducted for all babies with PNNJ at day 14	Full blood count and reticulocyte counts are conducted for all intermediate or high-risk babies and low risk babies if still jaundice at day 21. Full blood picture is considered only if there is a suspicion of ongoing or significant haemolysis (eg: low haemoglobin / pallor/ hepatosplenomegaly/ family history/ significant neonatal jaundice)	No more routine full blood picture in the workup for PNNJ.
8.	Assessment of stool colour by history or inspection	Not emphasised	Assessment of stool colour by history or inspection is emphasised.	Pale stool signifies obstructive jaundice [21].
9.	Is warning signs for serious conditions (especially biliary atresia) routinely given?	No	Yes	This is to create awareness and serves as a safe-netting mechanism.
10.	Follow-up plans for well babies who are still jaundice (low risk cases)	No. Babies are rendered heel-prick capillary bilirubin till jaundice resolves.	If day-21-tests were normal, the baby could be discharged with warning signs and reviewed during routine medical examination at 1 and 2 months old.	This will reduce unnecessary investigations, clinic visits and improve compliance to follow up.

Abbreviations: *T4* Thyroxine, *TSH* Thyroid-Stimulating Hormone, *PNNJ* Prolonged Neonatal Jaundice, *UTI* Urinary Tract Infection

In Phase III, post-interventional phase, an assessment was conducted in the 12 hospitals six months later. The same mechanism of data collection as in Phase I was repeated to evaluate 20 most recent PNNJ cases.

A self-reporting surveillance system was also created for paediatricians in the Perak state to report on any biliary atresia, urinary tract infection, or congenital hypothyroidism cases. Information, which included the age of the infant, when and how the diagnoses of those conditions were made, and whether the new protocol was used -- was emailed or faxed to the author. This reporting was more of a voluntary than a compulsory one and attempted to identify any cases missed after the implementation of the new protocol. A regional level PNNJ registry was also introduced and initiated by the Perak state health department to collect monthly returns of PNNJ cases from all government health facilities.

### Meeting and consensus among stakeholders

A total of 32 members consisting of paediatricians, public health professionals and local policy stakeholders (2 hospital directors, 6 paediatricians, 9 representatives from health district office/ health clinics, 1 pathologist, 1 laboratory technician, 5 hospital medical officers, 3 Clinical Research Centre, CRC, members, 5 staff nurses) met on 10th August 2012 to review the results of Phase I (see Results) and were guided by the findings of the literature review (see Additional file 2 Table S1, Additional file 3 Table S2, Additional file 4 Table S3, Additional file 5 Table S4 and Additional file 6 Table S5).

Several issues in the management of PNNJ were identified from the data analysis from Phase I when compared with the latest evidence. The differences between the current practice and new protocol are outlined in Table 1. A new protocol was developed to address these issues. The new protocol, which was agreed upon during the consensus meeting, consists of a management flow chart which is based on risk stratification (Fig. 2), assessment form (Fig. 3) and parental education on warning signs; to guide the medical officer at primary care clinics and hospital on the management of PNNJ. Babies with PNNJ are categorized into high risk (in the presence of lethargy/ septic, poor perfusion, respiratory distress or poor feeding); intermediate risk (in the presence of conjugated hyperbilirubinaemia, total serum bilirubin > 300 μmol/L, new onset of jaundice, pale stool, dark yellow urine, pallor, poor weight gain, hepatosplenomegaly, jaundice > 1 month not investigated before, significant family history, bottle fed > 50%, or other suspected medical condition); or low risk (without any features in the high or intermediate risk group).

### Implementation of the new protocol

At a Perak state meeting held on the 4th of September 2012, the new protocol was introduced to the state health director, health district directors, hospital directors, paediatricians, and nursing representatives. The new protocol was distributed statewide upon agreement of stakeholders. A state prolonged neonatal jaundice registry to monitor monthly returns on prolonged neonatal jaundice cases from all hospital was introduced.

### Data analysis

Data collected were entered into Microsoft Excel 2010 and SPSS version 15.0 was used to analyse the data collected. Numerical variables were calculated as mean and standard deviation, and independent t-test was employed to compare mean generated from data collected from pre- and post-intervention. Categorical data collected was presented in the form of frequency and percentages. A *p*-value less than 0.05 was deemed to be statistically significant.

All data collected was kept confidential, and no unique identifiers were collected for PNNJ cases. Data presented did not specifically identify any clinic or hospital that responded to this study.

The main ethical issue encountered before implementing the new protocol was the risk of not detecting serious conditions because total serum bilirubin with differentials was the only test screened in well, term breastfed babies. This risk was minimised with improved clinical assessment and warning signs education to all parents.

## Results

### The changes seen in practice after implementing the new protocol

A total of 240 cases were collected from 12 hospitals of Perak state; however, only data from 199 case notes pre-intervention and 145 case notes post-intervention with the new protocol were included for analysis. Cases with incomplete data or not fulfilling the study inclusion criteria were excluded. The data gathered were compared and shown in Table 2.

- Postnatal age upon referral

**Table 2:** Comparison of mean score of pre- and post-intervention

Management of prolonged neonatal jaundice	Mean (SD)		*p* value*
	Pre (*n* = 199 cases)	Post (*n* = 145 cases)	
Postnatal age upon referral (day)	16.54 (± 5.46)	20.01 (±11.14)	*p* = 0.001
Days taken to be seen at hospital level after referral (days)	20.9 (±11.38)	21.5 (±9.69)	*p* = 0.617
Clinical Assessment			
●5 important points in patient history taking (score)^a^	3.26 (±1.58)	4.44 (±0.92)	*p* < 0.001
●4 important points in family history taking (score)^b^	0.53 (±1.10)	2.14 (±1.89)	*p* < 0.001
●5 important points in physical examinations (score)^c^	3.78 (±1.50)	4.49 (±1.00)	*p* < 0.001
Number of lab investigations done before referral to the hospital	2.22 (±2.09)	1.57 (±1.68)	*p* = 0.020
Total number of laboratory investigations done per patient at the hospital level	9.01 (±2.99)	5.81 (±3.12)	*p* < 0.001
Total number of visits per patient from the time of referral to discharge	2.46 (±1.27)	2.20 (±0.92)	*p* = 0.040
Warning sign given^d^	NA	75.2%	NA

*Student T-test was used to compare mean score of managing PNNJ pre and post implementation of new protocol

^a^Patient history referring to feeding method, self-reported stool colour, urine colour, weight gain, neonatal jaundice (before day 14 of life)

^b^Family history referring to family history of blood disorders, severe/obstructive jaundice, renal problem, congenital hypothyroidism

^c^Physical examination referring to general appearance of the baby, respiratory, cardiovascular, gastrointestinal/ organomegaly and central nervous system

^d^Warning sign referring to unwell baby/ pale stool dark yellow urine/ new onset of jaundice/ persistent jaundice > 2 months

The mean postnatal age of babies upon referral has significantly increased from 16.54 days to 20.01 days (*p* = 0.001).

b. Clinical assessment of PNNJ

The average score for patient-related history taking, where the maximum achievable score was 5, had improved from 3.26 to 4.44 (*p* < 0.001). Mean score of family history taking, with a maximum score of 4, had increased from 0.53 to 2.14 (*p* < 0.001). The average physical examination score, with a maximum achievable score of 5, had risen from 3.8 to 4.5 (*p* < 0.001).

c. Number of laboratory Investigations done before referral to hospital

There was a significant reduction in the total number of investigations done before referral to the hospital (from 2.2 to 1.7, *p* = 0.020).

d. Number of laboratory investigations done at the hospital level

A total of 1758 tests were done in 199 patients pre-intervention compared to 811 tests in 145 patients post-intervention. The new protocol had significantly reduced the total number of laboratory investigations done per patient at the hospital level, from 9.01 laboratory investigations per patient to 5.81 per patient (*p* < 0.001).

e. Type of laboratory investigations done at the hospital level

The type and number of laboratory investigations done at the hospital level was more reasonable after the implementation of the new protocol (see Table 3). Pre-intervention, out of a total of 1758 tests, total serum bilirubin without differential was the most frequently done (249 tests), followed by urine culture and sensitivity test (237 tests), liver function test (198 tests), and total serum bilirubin with differentials (194 tests). Post-intervention, out of a total of 811 tests, the top four laboratory investigations done were total serum bilirubin with differentials (203 tests), urine dipstick & microscopy test (107 tests), free T4/TSH (105 tests), and full blood count (98 tests).

f. Total number of visits to the hospital

**Table 3:** The type and number of laboratory investigation pre- and post-intervention

Pre-intervention^e^		Post-intervention^f^	
Type of laboratory investigation	The number of laboratory investigations; *n* = 1758	Type of laboratory investigation	The number of laboratory investigations; *n* = 811
Total serum bilirubin without differential	249 (14.2)	Serum bilirubin with differential	203 (25.0)
Urine culture and sensitivity test	237 (13.5)	Urine dipstick & microscopy test	107 (13.2)
Liver function test	198 (11.3)	Free T4/TSH	105 (12.9)
Serum bilirubin with differential	194 (11.0)	Full blood counts	98 (12.1)
Free T4/TSH	180 (10.2)	Reticulocyte count	86 (10.6)
Full blood counts	165 (9.4)	Total serum bilirubin without differential	39 (4.8)
Full blood picture	139 (7.9)	Urine culture and sensitivity test	25 (3.1)
Urine dipstick & microscopy test	131 (7.5)	Liver function test	63 (7.8)
Reticulocyte count	102 (5.8)	Renal Profile	24 (3.0)
Renal Profile	87 (4.9)	Full blood picture	21 (2.6)
G6PD	47 (2.7)	G6PD	9 (1.1)
Blood Group	24 (1.4)	Blood Group	0 (0.0)
Ultrasound	3 (0.2)	Ultrasound	3 (0.4)
TORCHES	2 (0.1)	TORCHES	4 (0.5)
Urine dipstick	0 (0.0)	Urine dipstick	24 (3.0)

^e^199 patients were in pre-intervention phase

^f^145 patients were in post-intervention phase

Abbreviation: *T4* Thyroxine, *TSH* Thyroid-Stimulating Hormone, *G6PD* Glucose-6-phosphate dehydrogenase, *TORCHES* Toxoplasmosis, Rubella, Cytomegalovirus, Herpes, Syphilis

There was only a slight reduction of the mean number of total visits to the hospitals for PNNJ (from 2.46 to 2.20, *p* = 0.046).

### Self-reporting surveillance system

As of September 2013, the self-reporting system recorded 13 cases of urinary tract infection, 4 cases of congenital hypothyroidism, and 3 cases of suspected biliary atresia, which on further investigations were found to be all detected by the new protocol.

### Prolonged neonatal jaundice registry

Out of 9967 total live births, 1576 cases of prolonged neonatal jaundice were reported to the Prolonged Neonatal Jaundice Registry (Table 4). This gives a PNNJ incidence rate of 158 per 1000 live births. The majority of the cases were detected in Health Clinics (78.7%) and of all the cases detected, 92% were stratified as low risk, 4% as intermediate and 4% high risk (see Table 4).

**Table 4:** Incidence rate of PNNJ as recorded in Perak regional registry

Month	Total Live Birth, n	Total PNNJ Case, n (%)	Facility of Detection, n (%)		Risk Stratification, n (%)		
			Clinic	Hospital	Low	Intermediate	High
Jan	2436	430 (17.7)	325 (75.6)	105 (24.4)	413 (96.0)	13 (3.0)	4 (1.0)
Feb	1657	226 (13.6)	154 (68.1)	72 (31.9)	212 (94.0)	5 (2.0)	9 (4.0)
Mar	2681	494 (18.4)	396 (80.2)	98 (19.8)	445 (90.0)	20 (4.0)	29 (6.0)
Apr	3193	426 (13.3)	366 (85.9)	60 (14.1)	388 (91.0)	26 (6.0)	13 (3.0)
Total	9967	1576 (15.8)	1241 (78.7)	335 (21.3)	1450 (92.0)	63 (4.0)	63 (4.0)

## Discussion

### Principal findings

There was no standardized management of PNNJ among the government hospitals and health clinics in Perak state. In addition to this, clinical assessments were incomplete.

A new protocol for the management of PNNJ was developed, which focused on risk stratification by good clinical assessment. The flow chart and the assessment form aided the health care providers to perform better history taking and examination; to have a clear guide on the necessary laboratory investigations, referral indications, and follow-up plans; and to give warning signs to the parents. From the literature review, the four most useful investigations in the initial management of PNNJ were found to be total serum bilirubin with differentials, Free T4/TSH, urine dipstick and microscopy, and full blood count plus reticulocyte counts. These tests were adapted into the new protocol.

There were significant improvements in certain aspects of PNNJ management after implementation of the new protocol. Skills in patient history and family history taking as well as physical examination showed significant improvement after implementing the new protocol. The number of laboratory investigations done before referring to the hospitals and at the hospital level was significantly reduced.

With a clear guidance from the new protocol, many well breastfed babies with PNNJ were currently managed at the local health clinics, and only babies with concerns or risk factors were referred to the hospitals -- thus explaining the older postnatal age (by approximately four days) upon referral to hospitals post-intervention.

The choice of laboratory investigations for the initial management of PNNJ was also more rational post-intervention. Total serum bilirubin without differential, which is known to be unhelpful to determine the causes of PNNJ, was frequently done (294 times in 199 babies) before implementation of the new protocol. Post-intervention, this test was only done 39 times in 145 babies, a remarkable feat in diminishing the old practice. Of note, total serum bilirubin with differential was done at least twice the frequency of other tests post-intervention, reflecting the possibility that PNNJ in many babies resolved by the third week without warranting further tests.

We note that there was minimal reduction of the mean number of total visits to the hospitals. This small effect size could be explained by the fact that 9 out of 12 hospitals were non-specialist hospitals and post-intervention they still served as a primary care centres for nearby patients.

Follow-up plans were unclear in the previous system. Most babies underwent repeated heel-prick capillary bilirubin till jaundice resolved. The new protocol enabled well, low-risk babies with normal tests results at day 21 to be discharged with warning signs and to be reviewed only at one-month and two-month old routine medical examination (RME). The requirement for less visits reduced the burden to parents and health care facilities while being sufficiently safe and effective.

### Strengths and weaknesses of the study

This was the first study locally that looked into the burden and management of PNNJ, at both the primary care and tertiary care levels. In local terms, the strengths of the new protocol were standardising the approach to managing babies with PNNJ, empowering the health clinics to manage well babies, emphasising good clinical assessment, rationalizing the choice of investigations, giving guidance on how to follow-up well babies and creating a safety net by recommending warning signs for parents. Additionally, the state regional registry of prolonged neonatal jaundice had provided the incidence rate of PNNJ, which was not available nationally. The findings showed that prolonged neonatal jaundice is common and indirectly signifies a major workload.

The sampling method was the main limitation of this study. Case notes were conveniently selected by paediatricians during the pre-intervention phase, and this could result in sampling bias.

### Comparison to other studies

There were several studies of prolonged neonatal jaundice but was based from a single centre (Additional file 3: Table 2) [22–27]. Other studies looked at the incidence and management of certain conditions that were related to PNNJ (Additional file 4: Table S3) [26, 28, 29].

### Implications for clinicians and policymakers

Clinical assessment, including the inspection of stool colour, remains the most important aspect in the management of prolonged neonatal jaundice. The essential laboratory investigation needed at two weeks of life is the total serum bilirubin with differentials, and further workup is required:

- in well, low risk babies who remain jaundice at 3 weeks of life (total serum bilirubin with differentials, urine dipstick plus microscopy, Free T4/ TSH, full blood count plus reticulocyte counts), or
- in the presence of positive clinical findings, at any age (the laboratory investigations above plus other relevant tests eg: full blood picture, liver function test etc.).

The reduction of the number of blood investigations and clinic visits by risk stratification approach has potential in improving quality of care for babies, parents’ satisfaction and costs.

## Conclusion

A revised regional evidenced-based PNNJ management protocol had managed to use a risk stratification approach and successfully reduced the number of visits, investigations and improved the quality of care for neonates. This protocol has since been incorporated into the Ministry of Health national NNJ programme.

## Additional files

Additional file 1: Data collection form. (DOCX 26 kb)

Additional file 2: Table S1: A summary of local and international protocols of PNNJ management. (DOCX 38 kb)

Additional file 3: Table S2: Recommendations by other authors on the management of PNNJ. (DOCX 34 kb)

Additional file 4: Table S3: Studies related to the causes of PNNJ and their incidences. (DOCX 46 kb)

Additional file 5: Table S4: Comparison of American Association of Paediatrics and NICE guidelines in the management urinary tract infection (DOCX 21 kb)

Additional file 6: Table S5: Comparisons between other studies in the management of urinary tract infection in babies with jaundice. (DOCX 35 kb)

## Data Availability

The datasets generated and/or analysed during the current study are available in the Google Drive repository, https://drive.google.com/drive/folders/1U-uakDRkq5M9kujmqpGy%2D%2DRmQItRSPO5?usp=sharing

## Abbreviations

G6PD: Glucose-6-phosphate dehydrogenase
NNJ: Neonatal jaundice
PNNJ: Prolonged neonatal jaundice
T4: Thyroxine
TORCHES: Toxoplasmosis, Rubella, Cytomegalovirus, Herpes, Syphilis
TSH: Thyroid stimulating hormone
UK: United Kingdom
UTI: Urinary tract infection

## References

[1] Ng HP, Muhammad-Ismail H-I, Thomas T (2008). Paediatric protocols for Malaysian hospitals.

[2] Ng HP, Muhammad-Ismail H-I, Thomas T. Paediatric protocols for Malaysian hospitals. 3rd ed. Selangor Darul Ehsan, Malaysia, Ministry of Health Malaysia; 2012.

[3] Pot M, Sadler L, Hickman A, Baird T (2008). National Women’s Newborn Services Annual Clinical Report 2008.

[4] Ratnavel N, Ives NK (2005). Investigation of prolonged neonatal jaundice. Curr Paediatr.

[5] Maisels MJ, Clune S, Coleman K, Gendelman B, Kendall A, McManus S, Smyth M (2014). The natural history of jaundice in predominantly breastfed infants. Pediatrics.

[6] Clarkson JE, Cowan JO, Herbison GP (1984). Jaundice in full term healthy neonates--a population study. Aust Paediatr J.

[7] Fanaroff AA, Martin RJ, Walsh MC (2006). Fanaroff and Martin's neonatal-perinatal medicine : diseases of the fetus and infant.

[8] Kliegman R, Nelson WE (2007). Nelson textbook of pediatrics.

[9] Livesey E, Cortina Borja M, Sharif K, Alizai N, McClean P, Kelly D, Hadzic N, Davenport M (2009). Epidemiology of biliary atresia in England and Wales (1999-2006). Arch Dis Child Fetal Neonatal Ed.

[10] Fawaz R, Baumann U, Ekong U, Fischler B, Hadzic N, Mack CL, McLin VA, Molleston JP, Neimark E, Ng VL (2017). Guideline for the evaluation of Cholestatic jaundice in infants: joint recommendations of the north American Society for Pediatric Gastroenterology, hepatology, and nutrition and the European Society for Pediatric Gastroenterology, hepatology, and nutrition. J Pediatr Gastroenterol Nutr.

[11] Altman RP, Lilly JR, Greenfeld J, Weinberg A, van Leeuwen K, Flanigan L (1997). A multivariable risk factor analysis of the portoenterostomy (Kasai) procedure for biliary atresia: twenty-five years of experience from two centers. Ann Surg.

[12] Chardot C, Serinet MO (2006). Prognosis of biliary atresia: what can be further improved?. J Pediatr.

[13] Nio M, Ohi R, Miyano T, Saeki M, Shiraki K, Tanaka K (2003). Five- and 10-year survival rates after surgery for biliary atresia: a report from the Japanese biliary atresia registry. J Pediatr Surg.

[14] Serinet MO, Broue P, Jacquemin E, Lachaux A, Sarles J, Gottrand F, Gauthier F, Chardot C (2006). Management of patients with biliary atresia in France: results of a decentralized policy 1986-2002. Hepatology.

[15] Shneider BL, Brown MB, Haber B, Whitington PF, Schwarz K, Squires R, Bezerra J, Shepherd R, Rosenthal P, Hoofnagle JH (2006). A multicenter study of the outcome of biliary atresia in the United States, 1997 to 2000. J Pediatr.

[16] NICE: National Institute for health and care excellence. In: Neonatal jaundice*.* United Kingdom: NICE clinical guideline 98; 2010. 31886966

[17] Tan HS (2012). Assessing knowledge and diagnosis of prolonged neonatal jaundice among health care providers in Perak administrative region, Malaysia.

[18] National Collaborating Centre for Women's Children's health: National Institute for health and clinical excellence: guidance. In: Urinary Tract Infection in Children: Diagnosis, Treatment and Long-term Management. Edn. London: RCOG press National Collaborating Centre for Women's and Children's health.; 2007. 21290637

[19] Lee WS, Chai PF, Lim KS, Lim LH, Looi LM, Ramanujam TM (2009). Outcome of biliary atresia in Malaysia: a single-Centre study. J Paediatr Child Health.

[20] Wadhwani SI, Turmelle YP, Nagy R, Lowell J, Dillon P, Shepherd RW (2008). Prolonged neonatal jaundice and the diagnosis of biliary atresia: a single-center analysis of trends in age at diagnosis and outcomes. Pediatrics.

[21] Hsiao CH, Chang MH, Chen HL, Lee HC, Wu TC, Lin CC, Yang YJ, Chen AC, Tiao MM, Lau BH (2008). Universal screening for biliary atresia using an infant stool color card in Taiwan. Hepatology.

[22] Ogundele MO, Halliday J, Weir P (2010). Implementation of a prolonged neonatal jaundice protocol supported by electronic software. Clin Gov: Int J.

[23] Rodie ME, Barclay A, Harry C, Simpson J (2011). NICE recommendations for the formal assessment of babies with prolonged jaundice: too much for well infants?. Arch Dis Child.

[24] Rodie ME, Harry C, Taylor R, Barclay AR, Cochran D, Simpson JH (2012). Rationalized assessment of prolonged jaundice is safe and cost-effective. Scott Med J.

[25] Tyrell M, Hingley S, Giles C, Menakaya JO (2009). Impact of delayed screening for prolonged jaundice in the newborn. Arch Dis Child Fetal Neonatal Ed.

[26] Hannam S, McDonnell M, Rennie JM (2000). Investigation of prolonged neonatal jaundice. Acta Paediatr.

[27] Tizzard S, Davenport M (2007). Early identification and referral of liver disease. Community Pract.

[28] Crofts DJ, Michel VJ, Rigby AS, Tanner MS, Hall DM, Bonham JR (1999). Assessment of stool colour in community management of prolonged jaundice in infancy. Acta Paediatr (Oslo, Norway: 1992).

[29] Zakiah I, Zaini AR, Jamilah B, Zawiah A (1992). Alpha-1-antitrypsin deficiency in babies with prolonged jaundice. Malays J Pathol.

[30] Rennie J, Burman-Roy S, Murphy MS, Guideline Development G (2010). Neonatal jaundice: summary of NICE guidance. BMJ (Clin Res Ed).

[31] Soylu A. Relation between hyperbilirubinemia and urinary tract infections in the neonatal period. J Nephrol Ther. 2014;s11(01).

[32] Amar-Singh HSS. TSH screening of cord blood in Malaysia – its impact (the National Congenital Hypothyroid Screening Programme in Malaysian). J Endocrinol Metab. 2010;1(Issue 1, Supplement (May 2010).

