# Supplementary material for: Impact of a standardized protocol for the Management of Prolonged Neonatal Jaundice in a regional setting: an interventional quasi-experimental study
Source: BMC Pediatr. 2019 May 29;19:174. doi: 10.1186/s12887-019-1550-3 (PMC6540519; doi:10.1186/s12887-019-1550-3)
Supplement: Supplementary file 1 — Data collection form. (DOCX 26 kb) [file 12887_2019_1550_MOESM1_ESM.docx]

Additional File 1: Data Collection Form

| **Current Practice In Management Of Prolonged Jaundice In Perak State**  (Form to be filled by medical officers or paediatricians only)  **Date:**  Dear Colleague  We are carrying out this study to look into the current practice of management of prolonged jaundice in Perak state. The aim of this study is to improve the management of prolonged neonatal jaundice.  **Instructions:**   1. Please identify from your clinic the latest 20 infants who were referred for prolonged neonatal jaundice and have completed management. This will be retrospective data based on your clinic records. 2. Kindly fill in the relevant information below and tick in the boxes where appropriate. 3. We would be grateful if you complete the date forms within 2 week of receiving them. 4. All form filled must be supervised by a specialist if it is done by a medical officer. 5. Please return all completed data form to: **Dr. Inthira Sankari**   **Pediatric Department**  **Hospital Slim River**  **35800 Slim River, Perak**  **Contact No.: 05-4526494, Email:** [**shinthira@gmail.com**](mailto:shinthira@gmail.com)  **Inclusion criteria** (only include infants that fulfil this criteria):   1. **Term infants** 2. **Prolonged jaundice for more than 14 days of life** 3. **Birth weight more than 2.5 kg** | | | | | |
| --- | --- | --- | --- | --- | --- |
| 1. | Name of hospital | |  | | |
| 2. | Day of referral (day of life of neonate)  This is based on the date recorded on the referral letter | | Day…… | | |
| 3. | Day of assessment by medical officer or paediatrician after referral (day of life of neonate)  This is the date the child was first seen at your hospital for PNNJ as recorded in your clinical card. | | Day…… | | |
| 4. | Investigations done for prolonged jaundice workup before referral to your hospital | | \| **Please tick on the appropriate investigation done:** \| \| \| \| \| --- \| --- \| --- \| --- \| \| **Investigations** \| \| \| \| \| Full Blood Count (FBC) \|  \| Urine Full Examination & Microscopy Examination (UFEME) \|  \| \| Full Blood Picture (FBP) \|  \| Urine Culture & Sensitivity \|  \| \| Reticulocyte count \|  \| Urine dipsticks for leucocytes \|  \| \| Coombs test \|  \| Toxoplasmosis, Rubella, Cytomegalovirus, Herpes, Syphilis (TORCHES) \|  \| \| Blood group \|  \| Thyroxine / Thyroid stimulating hormone (T4/TSH) \|  \| \| Prothrombin Time/Activated Partial Thromboplastin Time \|  \| Inborn Errors of Metabolism (IEM) SCREEN \|  \| \| Glucose-6-phosphate dehydrogenase (G6PD) \|  \| Alpha antitrypsin \|  \| \| Renal Profile \|  \| Galactose-1-phosphate Uridyltransferase (GAL-1-PUT) test \|  \| \| Total Serum Bilirubin \|  \| Ultrasound abdomen \|  \| \| Direct /Indirect Serum bilirubin \|  \| Hepatobiliary Iminodiacetic Acid (HIDA) scan \|  \| \| Liver function test \|  \| Liver biopsy \|  \| \| If other test, please state: \| \| \| \| | | |
| 5. | What history was taken | |  | | |
|  | Feeding method | | Yes 🞎 | No 🞎 | |
|  | Color of stool | | Yes 🞎 | No 🞎 | |
|  | Color of urine | | Yes 🞎 | No 🞎 | |
|  | Weight gain | | Yes 🞎 | No 🞎 | |
|  | Neonatal jaundice (before Day 14 of life) | | Yes 🞎 | No 🞎 | |
|  |  | | *No=Not done or not documented | | |
| 6. | Was a history taken on the family risk factors | |  |  | |
|  | Family history of blood disorders | | Yes 🞎 | No 🞎 | |
|  | Severe / obstructive jaundice | | Yes 🞎 | No 🞎 | |
|  | Renal problem | | Yes 🞎 | No 🞎 | |
|  | Congenital hypothyroidism | | Yes 🞎 | No 🞎 | |
|  |  | | *No=Not done or not documented | | |
| 7. | | Was a physical examination was done? |  | |  |
|  |  | General appearance | Yes 🞎 | | No 🞎 |
|  |  | Respiratory | Yes 🞎 | | No 🞎 |
|  |  | Cardiovascular | Yes 🞎 | | No 🞎 |
|  |  | Gastrointestinal / organomegaly | Yes 🞎 | | No 🞎 |
|  |  | Central nervous system | Yes 🞎 | | No 🞎 |
|  |  |  | *No=Not done or not documented | | |
| 8 | | Investigations done for prolonged jaundice workup at **first presentation** at your hospital | \| **Please tick on the appropriate investigation done:** \| \| \| \| \| --- \| --- \| --- \| --- \| \| **Investigations** \| \| \| \| \| Full Blood Count (FBC) \|  \| Urine Full Examination & Microscopy Examination (UFEME) \|  \| \| Full Blood Picture (FBP) \|  \| Urine Culture & Sensitivity \|  \| \| Reticulocyte count \|  \| Urine dipsticks for leucocytes \|  \| \| Coombs test \|  \| Toxoplasmosis, Rubella, Cytomegalovirus, Herpes, Syphilis (TORCHES) \|  \| \| Blood group \|  \| Thyroxine / Thyroid stimulating hormone (T4/TSH) \|  \| \| Prothrombin Time/Activated Partial Thromboplastin Time \|  \| Inborn Errors of Metabolism (IEM) SCREEN \|  \| \| Glucose-6-phosphate dehydrogenase (G6PD) \|  \| Alpha antitrypsin \|  \| \| Renal Profile \|  \| Galactose-1-phosphate Uridyltransferase (GAL-1-PUT) test \|  \| \| Total Serum Bilirubin \|  \| Ultrasound abdomen \|  \| \| Direct /Indirect Serum bilirubin \|  \| Hepatobiliary Iminodiacetic Acid (HIDA) scan \|  \| \| Liver function test \|  \| Liver biopsy \|  \| \| If other test, please state: \| \| \| \| | | |
| 9 | | **Total number of investigations done** for prolonged jaundice workup in hospital and clinic | \| Total number of investigations:………………. \| \| \| \| \| \| --- \| --- \| --- \| --- \| --- \| \| Please state the **number** of investigations done: \| \| \| \| \| \| **Please tick on the appropriate investigation done:** \| \| \| \| \| **Investigations** \| \| \| \| \| Full Blood Count (FBC) \|  \| Urine Full Examination & Microscopy Examination (UFEME) \|  \| \| Full Blood Picture (FBP) \|  \| Urine Culture & Sensitivity \|  \| \| Reticulocyte count \|  \| Urine dipsticks for leucocytes \|  \| \| Coombs test \|  \| Toxoplasmosis, Rubella, Cytomegalovirus, Herpes, Syphilis (TORCHES) \|  \| \| Blood group \|  \| Thyroxine / Thyroid stimulating hormone (T4/TSH) \|  \| \| Prothrombin Time/Activated Partial Thromboplastin Time \|  \| Inborn Errors of Metabolism (IEM) SCREEN \|  \| \| Glucose-6-phosphate dehydrogenase (G6PD) \|  \| Alpha antitrypsin \|  \| \| Renal Profile \|  \| Galactose-1-phosphate Uridyltransferase (GAL-1-PUT) test \|  \| \| Total Serum Bilirubin \|  \| Ultrasound abdomen \|  \| \| Direct /Indirect Serum bilirubin \|  \| Hepatobiliary Iminodiacetic Acid (HIDA) scan \|  \| \| Liver function test \|  \| Liver biopsy \|  \| \| If other test, please state: \| \| \| \| | | |
| 10. | | **Total** number of visits from time of referral to discharge | **__________** | | |
| 11. | | Final Diagnosis |  | |  |
|  |  | Breast milk jaundice | Yes 🞎 | | No 🞎 |
|  |  | Biliary atresia | Yes 🞎 | | No 🞎 |
|  |  | Congenital hypothyroidism | Yes 🞎 | | No 🞎 |
|  |  | Hereditary blood disorders | Yes 🞎 | | No 🞎 |
|  |  | Urinary tract infection | Yes 🞎 | | No 🞎 |
|  |  | Not stated | Yes 🞎 | |  |
|  |  | Other causes | Please state …………………… | | |
| 12. | | Plan | Discharge | | 🞎 |
|  |  |  | Follow up | | 🞎 |
|  |  |  | For admission | | 🞎 |
|  |  |  | Refer to sub-speciality | | 🞎 |
| **The End. Thank you** | | | | | |
