# Supplementary material for: Impact of a standardized protocol for the Management of Prolonged Neonatal Jaundice in a regional setting: an interventional quasi-experimental study
Source: BMC Pediatr. 2019 May 29;19:174. doi: 10.1186/s12887-019-1550-3 (PMC6540519; doi:10.1186/s12887-019-1550-3)
Supplement: Supplementary file 2 — Table S1: A summary of local and international protocols of PNNJ management. (DOCX 38 kb) [file 12887_2019_1550_MOESM2_ESM.docx]

# Additional file 2: Table S1: A summary of local and international protocols of PNNJ management

| **No** | **Local & International**  **Guidelines** | **Year** | **Definition/**  **Diagnosis of PNNJ** | **History / Physical Examination** | **Level of Care**  **Mentioned** | **Investigations** | | | | | | | | | | | **Comments** |
| --- | --- | --- | --- | --- | --- | --- | --- | --- | --- | --- | --- | --- | --- | --- | --- | --- | --- |
|  |  |  |  |  |  | **Type** | Lists | | | | | | | | | |  |
|  |  |  |  |  |  |  | A | B | C | D | E | F | G | H | I | J |  |
| 1 | Ng HP, Hussain-Imam HMI, Thomas T: Paediatric Protocols For Malaysian Hospitals 3rd Edition [[2](#_ENREF_2)]. | 2012 | > 2 weeks old in term  OR > 3 weeks old in preterm infant    Visible jaundice  (Serum bilirubin> 85µmol/L)    Conjugated: > 25µmol/L | Not mentioned    *Early diagnosis of biliary atresia and hypothyroidism is important for favourable long-term outcome. | Not mentioned  Unconjugated  hyperbilirubinaemia: to exclude UTI and hypothyroidism.    Admit if unwell.  Follow-up till jaundice resolves. | Standard list for both unconjugated and conjugated hyper-  bilirubinaemia | ❶ | ❷ | ❸ | ❹ | ❺R | ❻ |  |  | ❼T | ❽A | Breast milk jaundice: a diagnosis of exclusion → babies must be well, gaining weight, exclusively breastfed and stool is yellow.    Very useful section on causes and investigations for conjugated hyperbilirubinaemia & neonatal hepatitis |
| 2 | Malaysia Ministry of Health –  Integrated plan for the detection and  management of  neonatal jaundice (2^nd^ edition) [[24](#_ENREF_24)] | 2008 | > 2 weeks old in term infant  OR > 3 weeks old in preterm infant  Visible jaundice  (Serum bilirubin > 85µmol/L)  Conjugated: >34µmol/L or >15% | Not mentioned | Conjugated  hyperbilirubinaemia must be referred to paediatric department urgently.  Those with unconjugated can be investigated first and referred if jaundice not resolved or a definitive cause found. | Standard list for unconjugated hyper- bilirubinaemia | ❶ | ❷ | ❸ | ❹ | ❺R | ❻ |  |  | ❼T |  | Breast milk jaundice Babies must be well, gaining weight, exclusively breastfed and stool is yellow. Management: continue breastfeeding  Causes and investigations for conjugated hyperbilirubinaemia are mentioned |
| 3 | NICE clinical guidelines. Neonatal Jaundice [[25](#_ENREF_25)]. | 2010 | > 2 weeks old in babies > 37 weeks.  OR > 3 weeks old in babies < 37 weeks. | Mentioned about pale chalky stools/ dark urine. | Not mentioned | Standard list | ❶ | ❷ | ❸ | ❹ | ❺ |  | ❻ | ❼ |  |  | Conjugated bilirubin > 25µmol/L |
| 4 | American Academy of Pediatrics – Management of  hyperbilirubinemia  in infants > 35 weeks of gestation [[29](#_ENREF_29)] | 2004 | Jaundice > 3 weeks, conjugated bilirubin >20%. | Evaluate for signs and symptoms of hypothyroidism. | Not mentioned | Standard list | ❶^ | ❷^ |  |  |  |  |  |  |  |  | To investigate for causes of cholestasis if direct bilirubin elevated |
| 5 | South Australian  Perinatal Practice  Guidelines. Neonatal Jaundice [[27](#_ENREF_27)] | 2010 | > 2 weeks old in babies > 35 weeks.  OR > 3 weeks old in babies < 35 weeks. | Acholic stool require prompt review. | Not mentioned | Standard list | ❶ | ❷ | ❸ | ❹ |  |  |  |  |  |  | Breast-milk jaundice will resolve over 2-3 months.  Do not recommend stop breastfeeding |
| 6 | New Zealand– Newborn Services  Guidelines and Protocols. Assessment of Prolonged and Late-Onset Jaundice [[26](#_ENREF_26)] | 2004 | > 2 weeks old in term infants,  OR > 3 weeks old in preterm infants. | Prolonged neonatal jaundice in artificially-fed infants should be aggressively investigated.  Jaundice only appears after day 7 should be investigated carefully.    Assessment of growth/ feeding/ examination of stool colour and enquiry about urine colour should be done. | Parents asked to contact General Practitioner or Local medical committee if they have concerns (when investigation are normal). | Standard list | ❶ | ❷ | ❸ | ❹ | ❺ | ❻ |  | ❼* | ❽ |  | 9% of breastfed babies are still jaundice at 28 days    Newborn screening test in New Zealand evaluates TSH    Bag urine sample may yield ambiguous results which may require further test (potentially invasive or unnecessary)    Liver function test only if conjugated  hyperbilirubinaemia.    Do not recommend to stop breastfeeding |
| 7 | Royal Children's  Hospital,  Melbourne,  Australia [[28](#_ENREF_28)] | 2008 | > 2 weeks old in term infant  OR > 3 weeks old in preterm infant | The onset of jaundice, sign of dehydration /poor weight gain, birth trauma, significant bruising, maternal history, family history of haemolytic disease, dark urine or pale stools should be obtained. | DWED senior or  Paediatrician for  prolonged neonatal jaundice | According to  unconjugated or  conjugated hyper- bilirubinaemia |  | ❶ | ❷ | ❸ | ❹ |  |  | ❻ | ❼ | ❽A | Flowchart which encompasses all babies  with jaundice    If all normal, to arrange for follow-up and ensure  feeding well |
| 8 | Israel Guidelines –  Preliminary approach to infants with PNNJ [[23](#_ENREF_23)]. | 2008 | > 10-14 days old | Gestational age, history of jaundice in siblings, feeding, success of breastfeeding, weight gain, and physical examination should be obtained. | Not mentioned | Standard list |  | ❶ | ❷ | ❸ |  |  | ❹ | ❺ |  | ❻A | Liver function test if conjugated hyperbilirubinaemia. If persistent conjugated hyperbilirubinaemia, abdomen ultrasound and investigation for cholestatic jaundice are needed. |
| **Summary:**   1. The American Academy of Pediatrics [[29](#_ENREF_29)] and South Australian guidelines [[27](#_ENREF_27)] included babies >35 weeks in their discussion for term babies. 2. Except for the Malaysian [[1](#_ENREF_1), [2](#_ENREF_2)] and the Melbourne Royal Children Health protocols [[28](#_ENREF_28)], all others protocols mentioned a few points on clinical assessment. 3. Of note, the New Zealand guidelines [[26](#_ENREF_26)] touches most on clinical assessment, also mentioned that: Prolonged neonatal jaundice in artificially-fed babies should be aggressively investigated, jaundice appears only after day 7 should be investigated carefully, and there should be assessment of growth, feeding, examination of stool colour and enquiry about urine colour 4. All national protocols have a standard list of investigations for neonates with prolonged neonatal jaundice, irrespective of clinical assessment. Out of these, 3 has a standard list for unconjugated hyperbilirubinaemia [[1](#_ENREF_1), [2](#_ENREF_2), [24](#_ENREF_24)]. 5. The standard list of investigations includes 6-8 different tests, except American Academic of Pediatrics (only 2 tests)[[29](#_ENREF_29)]. 6. All protocols recommended total and differential bilirubin as part of investigations 7. Only 4 out of 8 protocols mentioned the level of care for the management of prolonged neonatal jaundice [[2](#_ENREF_2), [24](#_ENREF_24), [26](#_ENREF_26), [28](#_ENREF_28)]. | | | | | | | | | | | | | | | | | |
| **Conclusion:**  Across most local and international protocols, the focus was more on a standard list of investigations (many tests) rather than on the clinical assessment. Only a few mentioned the level of care required for this group of babies. Flow charts are difficult to obtain for prolonged neonatal jaundice. | | | | | | | | | | | | | | | | | |

| **Legend** |  |
| --- | --- |
| A: Review Newborn Screening Test | F: Full blood picture |
| B: Total and differential/ conjugated bilirubin | G: Mother's blood group & Baby's blood group |
| C: Free T4/ TSH | H: Coombs' test |
| D: Urine Culture +/- microscopy | I: G6PD test (in selected babies) |
| E: Full blood count | J: Others (a – urine reducing substances, b – liver function test) |
| ❶❷❸ Number of investigations recommended |  |
|  |  |
| * : only if baby anaemic, having early jaundice and evidence of haemolysis on full blood count | |
| ^ : evidence quality D (benefit versus harms exceptional) | |
| R: includes reticulocyte count | |
| T: Trace | |
