# Supplementary material for: Impact of a standardized protocol for the Management of Prolonged Neonatal Jaundice in a regional setting: an interventional quasi-experimental study
Source: BMC Pediatr. 2019 May 29;19:174. doi: 10.1186/s12887-019-1550-3 (PMC6540519; doi:10.1186/s12887-019-1550-3)
Supplement: Supplementary file 3 — Table S2: Recommendations by other authors on the management of PNNJ. (DOCX 34 kb) [file 12887_2019_1550_MOESM3_ESM.docx]

# Additional file 3: Table S2: Recommendations by other authors on the management of PNNJ

| **No.** | **Institution/ Hospital Protocols** | **Authors & Title** | **Definition/ Diagnosis of PNNJ** | **Clinical Assessment of PNNJ** | **Level of Care**  **Mentioned** | **Investigations** | | | | | | | | | | | **Results/ Conclusion** |
| --- | --- | --- | --- | --- | --- | --- | --- | --- | --- | --- | --- | --- | --- | --- | --- | --- | --- |
|  |  |  |  |  |  | **Type** | **List** | | | | | | | | | |  |
|  |  |  |  |  |  |  | **A** | **B** | C | D | **E** | **F** | **G** | **H** | **I** | **J** |  |
| 1 | King's College Hospital, London  2000 | Hannam S *et al*. Investigation of Prolonged neonatal jaundice [[48](#_ENREF_48)]. | > 14 days old. | Prospective study of term babies referred to neonatal unit over 18 month’s period.  Inspection of colour of stool. | Yes – Paediatrician | Standard list |  | ❶  ① | ❷  ② | ❸  ③ | ❹  ④ |  | ⑤ | ⑥ | ❺  ⑦ | ⑧b | 154 out of 7139 live births had PNNJ    9 out of 154 babies were referred to subspecialty (1 giant cell hepatitis, 1 hepatoblastoma, 1 trisomy 9p, 2 UTI, 3 G6PD deficiency, 1 failure to regain body weight.) |
| 2 | Prolonged neonatal jaundice clinic at Hillingdon Hospital, Middlesex, United Kingdom  2009 | M Tyrell, S Hingley, C Giles, *et al*.  Letters - Impact of delayed screening for prolonged neonatal jaundice in the newborn [[32](#_ENREF_32)]. | > 14 days old. | Referred at day 14, detailed clinical assessment carried out at least at D21 by Paediatrician.  43.2% not jaundiced, 51.3% (94/183) jaundiced by Day 21. 16/ 94 had pathology. | Yes - Paediatrician | Standard list for jaundiced group of babies |  | ❶ | ❷ | ❸ | ❹ | ❺ | ❻ | ❼ | ❽ | ❾❿a, b | 80% discharged after one visit, 18% seen at 3 weeks and 40% seen at 2 weeks were recalled.  No biliary atresia was identified.  Conclusion: Delayed assessment reduced significantly the number of babies requiring screening test without missing any important pathologies. |
| 3 | Hampshire Community Health Care  2009 | Liz Taylor. Guidelines on Management of Prolonged neonatal jaundice (draft) [[52](#_ENREF_52)] | > 2 weeks old in term infant  OR > 3 weeks old in preterm babies | Role of Child Health Team.   - review weight/ general condition - assess feeding - inspect stool/ urine colour | Yes - Paediatrician | Based on flow chart |  | ❶ |  |  |  |  |  |  |  |  | Refer paediatrician/ general practitioner if babies:  - unwell  - not progressing normally  - abnormal stool/ urine colour  - conjugated bilirubin > 20% |
| 4 | Royal United Hospital, Bath  2008 | Ogundele MO, Halliday J, Weir P. A Rationalised protocol of selective screening for babies with prolonged neonatal jaundice [[22](#_ENREF_22)]. | >2 weeks of age in term babies and 3 weeks in pre-term babies. | Detailed clinical assessment of risk factors and limited screening tests for babies with prolonged neonatal jaundice. | Yes – general practitioners and paediatricians. | Selective screening. |  | ❶ |  |  |  |  |  |  |  |  | No serious liver disease was missed over two years. |
| 5 | Children's Liver Disease Foundation – Jaundice Protocol  2007 | Tizzard S, Davenport M. Early identification and referral of liver disease in infants [[49](#_ENREF_49)]. | Jaundice persisting | Important areas: feeding history, weight, document stool and urine colour | Health visitor/ midwife does general assessment and split bilirubin test  Refer Paediatrician if baby unwell/ conjugated hyperbilirubinaemia | Selective screening |  | ❶ |  |  |  |  |  |  |  |  | Recommended that all babies with prolonged neonatal jaundice be given a split bilirubin test. (No mentioning about other tests)  If unconjugated, then testing total serum bilirubin weekly until resolution. |
| 6 | Royal Hospital for Sick Children, Glasgow, United Kingdom  2011/ 2012 | ME Rodie *et al*. NICE recommendations for the formal assessment of babies with prolonged neonatal jaundice: too much for well infants? Postscript in Arch Dis Child 2011; 96: 111-112 [[30](#_ENREF_30)]  Published: Rationalised assessment of prolonged neonatal jaundice is safe and cost-effective. Scottish Medical Journal. 57 (3): 144-7, 2012Aug [[31](#_ENREF_31)]. | Beyond 14 days of life in a term baby and beyond 21 days in a preterm baby. | History, physical examination, stool inspection and split bilirubin was performed over two-year period in two level 3 neonatal units.  No significant pathology associated with prolonged neonatal jaundice was detected. | Neonate referred from community to Hospital neonatal unit. Reviews were performed by middle grade doctors with consultant readily available for advice | Rationalised approach to investigation derived from British Society of Paediatric Gastroenterology, Hepatology and Nutrition [[53](#_ENREF_53)] and Children’s Liver Disease Foundation [[54](#_ENREF_54)] |  | ❶  ① | ② | ③ | ④ |  | ⑤ |  | ❷  ⑥ | ⑦⑧ a, b | N=197 (1.5% of total live births of 12986 babies) presented as prolonged neonatal jaundice.  Number of repeat investigations (37 vs 7, *p*< 00001) and return appointments (28 vs 7, *p*= 0.0009) fell following the introduction of rationalised investigation algorithm. |
| **Summary:**   1. These were hospital or unit-based protocols on prolonged neonatal jaundice, mainly in United Kingdom looking into other options in deciding the types of investigations. 2. Of note, clinicians from Royal United Hospital in Bath (2008) and those From Royal Hospital for Sick Children in Glasglow (2011) had recommended a rational approach to the types of investigations, that is to base clinical decisions on the risk factors gathered from history and physical examination. The main test would only be the total and differential bilirubin. The authors from Bath also used computer software to assist in the assessment and management on this group of babies. Both centres felt, till the date of publication, risk stratification was a safe method [[22](#_ENREF_22)]. 3. The authors from Hillingdon Hospital, Middlesex, had also found that almost half of the term babies that were jaundice at 2 weeks of age were no more jaundice at 3 weeks old. Investigating these babies at 3 weeks, rather than 2 weeks had been a safe method while reducing visits and recalls [[32](#_ENREF_32)]. | | | | | | | | | | | | | | | | | |
| **Interpretation:**  The most important test in the management of PNNJ is the total serum bilirubin with differentials. Two ways were recommended to reduce tests and visits - that is to base the other tests on risk factors, or to investigate only at 3 weeks of life. | | | | | | | | | | | | | | | | | |

| **Legends** |  |
| --- | --- |
| A: Review Newborn Screening Test | F: Full blood picture |
| B: Total and differential/ conjugated bilirubin | G: Mother's blood group & Baby's blood group |
| C: Free T4/ TSH | H: Coombs' test |
| D: Urine Culture +/- microscopy | I: G6PD test (in selected babies) |
| E: Full blood count | J: Others (a – urine reducing substances, b – liver function test) |
| ❶❷❸ Number of investigations recommended | ①②③ Number of investigations previously practiced. |
|  |  |
| * : only if baby anaemic, having early jaundice and evidence of haemolysis on full blood count | |
| ^ : evidence quality D (benefit versus harms exceptional) | |
| R: includes Reticulocyte count | |
| T: Trace | |
