# Supplementary material for: Impact of a standardized protocol for the Management of Prolonged Neonatal Jaundice in a regional setting: an interventional quasi-experimental study
Source: BMC Pediatr. 2019 May 29;19:174. doi: 10.1186/s12887-019-1550-3 (PMC6540519; doi:10.1186/s12887-019-1550-3)
Supplement: Supplementary file 4 — Table S3: Studies related to the causes of PNNJ and their incidences. (DOCX 46 kb) [file 12887_2019_1550_MOESM4_ESM.docx]

# Additional file 4: Table S3: Studies related to the causes of PNNJ and their incidences

| **Incidence of prolonged neonatal jaundice in term and preterm babies** | | | | | |
| --- | --- | --- | --- | --- | --- |
| **No*.*** | **Authors** | **Incidence of PNNJ** | **Breastfeeding** | **Others** | |
| 1 | Hannam 2000 (London) [[48](#_ENREF_48)] | 154/ 7139 (2.1%) | 15-40% of breastfed babies | 1/ 154 had conjugated hyperbilirubinaemia | |
| 2 | Crofts 1999 (Sheffield) [[50](#_ENREF_50)] | 127/ 3661 (3.5%) | 125 / 127 were breastfed | -- | |
| 3 | Zakiah 1992 (HKL) [[51](#_ENREF_51)] | Not studied | Not studied | Over 3 years, there are 92/310 (29%) of conjugated babies with alpha-1-antitrypsin deficiency. | |
| **Biliary atresia – easily missed?** | | | | | |
|  | **Authors** | **Year studied** | **Age of diagnosis** | **No. of biliary atresia/ Clinical features** | **Others** |
| 1 | Lee WS [[19](#_ENREF_19)] | 1996-2005 (University Malaya) | Median age at referral of biliary atresia (n=57) was 62 days. | 3 most common causes of cholestasis among the 146 babies with neonatal cholestasis:   - Idiopathic neonatal hepatitis (n = 63, 43%), - Biliary atresia (n = 35, 24%) - Congenital cytomegalovirus hepatitis (n = 13, 9%).   Common clinical features at presentation were jaundice (100%), hepatomegaly (95%), splenomegaly (52%) and pale stools (47%).  3 clinical features noted to be sensitive for biliary atresia were   1. Presence of acholic or variably acholic stools on admission (sensitivity 77%, specificity: 51%) 2. Liver which was firm/hard in consistency (sensitivity: 77%, specificity 65%) 3. Palpable liver of ≥ 4 cm (sensitivity 94%, specificity: 39%) | 43% of biliary atresia patients encountered delay / had an inappropriate action taken before referral. Factors:   1. Repeated reassurances (n = 17, 26%) 2. Failure of hospital services at the referring hospital (n = 7, 11%) 3. Parental refusal for referral (n = 5, 8%)   2 years survival rate with native liver for the 48 patients who had Kasai was 37% (compares favourably with international figures), while the overall survival (native liver and liver transplant) rate was 40% (adversely affected by lack of timely liver transplant).  Author recommendations: The outcome of biliary atresia in **Malaysia** may be further improved by   1. Increasing the awareness on the importance of early referral for surgery in babies suspected of biliary atresia. 2. Repeated inspection of stools colour is necessary 3. Health authorities in Malaysia should consider the feasibility of universal stool colour screening in newborn babies to improve the outcome of biliary atresia. |
| 2 | Wadhwani SI, *et al* 2008  (Missouri, US) [[20](#_ENREF_20)] | 1990 – 2004 | Median age: 60 days  (21-152), | 55 | Worse in the recent era (2000-2004)  Attributed to neonatal follow-up practice |
| 3 | Lim CT, *et al* 1997 (Malaysia) [[33](#_ENREF_33)] | 1982 – 1991 | Mean age 20 days (  SD ± 17 d) | 35 | Same period 58 babies with neonatal hepatitis |
| 4 | Palermo JJ, *et al* 2012 (St Louis, Missouri US) [[55](#_ENREF_55)] | 29-item survey to community-based paediatricians, 108/230 (47%) of eligible physicians responded. First routine visit usually occurred in the 1^st^ week after discharge, 25% reported the 2nd visit was routinely scheduled after 4 weeks of age.  94% reported they would obtain a fractionated bilirubin for babies jaundiced beyond 4 weeks of age. If cholestasis identified at 6 weeks of age, 32% would obtain additional testing without referral to a subspecialist.  Conclusion: Management of early and prolonged neonatal jaundice is variable. The current practices appear to miss opportunities for early diagnosis of cholestasis and referral that are unlikely to be addressed without redesigning systems of care. | | | |
| 5 | Chen SM, *et al* 2005  (Taiwan) [[56](#_ENREF_56)] | 2002-2003, Infant stool colour card, attached to child health booklet. Extensive nationwide promotion to health care on stool colour.  Medical staff check with parents at 1 month old and cards returned to national infant stool colour card registry.  78184 (65%) of babies cards were returned, 29 out of 30 diagnosed with biliary atresia from stool colour card (26/29 before 60 days old). (Incidence biliary atresia 3.7/ 10 000) (Previously only 23% of BA picked up by 60 days) Yet 26% did not receive Kasai before 60 days.  Sensitivity: 89.7%, specificity: 99.9%, positive predictive value: 28.6% | | | |
| 6 | Lin YC, *et al* 2011  (Taiwan) [[57](#_ENREF_57)] | 2004–2009 in Taiwan: 185 biliary atresia. 2004–2006 (1.79 cases per 10 000 live births), 2007–2009 (1.23 cases per 10 000 live births) was decreased significantly (p=0.01).  Biliary atresia incidences were negatively correlated with the gross domestic product (p = 0.02) and marginally negatively correlated with rotavirus vaccine coverage rates (p = 0.07). | | | |
| 6 | Nio M, *et al* 2013  (Japan) [[58](#_ENREF_58)] | 1989-1999 | Only 505 (43%) underwent op < 60 days | | |
| **When does serum bilirubin level of a neonate fall to adult level?** | | | | | |
|  | **Authors** | **Statement/ Findings** | | | |
| 1 | Dennery 2001 (United Kingdom) [[59](#_ENREF_59)] | Serum bilirubin decline during the next several weeks | | | |
| 2 | Fenton 1998 [[60](#_ENREF_60)] | Time period inexact, although 14 days is commonly accepted as a cut-off point for investigation of sustained jaundice | | | |
| **What percentage of congenital hypothyroidism is missed in the Guthrie test/ Congenital Hypothyroidism Screening programme?** | | | | | |
|  | **Authors** | **Statement/ Findings** | | | |
| 1 | Hulse 1980 [[61](#_ENREF_61)] | TSH assay is effective in identifying mild cases of primary hypothyroidism but unable to detect secondary hypothyroidism. | | | |
| 2 | Grant 1988 (UK) [[62](#_ENREF_62)] | 493 out of 1,941,146 of congenital hypothyroidism (1: 3937). 4 cases missed for every 2 million | | | |
| 3 | Holtzman 1986 (United States) [[63](#_ENREF_63)] | 2 cases missed for every 1 million | | | |
| 4 | Amar Singh 2010  (Malaysia) [[35](#_ENREF_35)] | More than 85% of all births are covered by the national newborn screening program (95% of government births)  Birth prevalence of congenital hypothyroid is 1:3390. From 2001-2009, 576 neonates were confirmed to have congenital hypothyroidism by the screening programme.  The mean duration from birth to treatment was 11.6 days, with 90% treated within 20 days. | | | |
| 5 | Fuziah 2010 (Malaysia) [[34](#_ENREF_34)] | However, even experienced screening programs are not perfect as about 8 -10% of cases is still missed | | | |
| 6 | Zarina 2008 (Malaysia) [[64](#_ENREF_64)] | From the audit over a period of 25 months, a total of 13,875 newborn babies were screened with a coverage of 98.8%, 2 had congenital hypothyroidism. | | | |
| **Summary:**   1. Different authors had quoted the incidence rate of prolonged neonatal jaundice at around 2-4% [[48](#_ENREF_48), [50](#_ENREF_50), [51](#_ENREF_51)]. 2. Late diagnosis of biliary atresia remained a problem in many countries. In the United States, according to Wadhwani SI, *et al* the median age of diagnosis was 60 (21-152 days) because of neonatal follow-up practice [[20](#_ENREF_20)]. 3. Locally, in 1997, CT Lim had quoted the mean age of diagnosis of biliary atresia was 20 days. In 2008, WS Lee reported that the median age of referral for biliary atresia was 62 days [[33](#_ENREF_33)]. 4. WS Lee had found that common clinical features at presentation for biliary atresia were jaundice (100%), hepatomegaly (95%), splenomegaly (52%) and pale stools (47%). 3 sensitive markers were: 1) acholic or variably acholic stools on admission, 2) firm/ hard liver; and 3) palpable liver of >=4 cm, though the specificity were poor [[19](#_ENREF_19)]. 5. Taiwan’s national stool colour chart registry had successfully brought down the age of diagnosis of biliary atresia [[21](#_ENREF_21), [56](#_ENREF_56)]. 6. As for congenital hypothyroidism, the screening at birth will still miss secondary hypothyroidism. Locally, there are still a fraction of babies not screened by the national screening programme for congenital hypothyroidism [[34](#_ENREF_34), [35](#_ENREF_35), [64](#_ENREF_64)]. | | | | | |
| **Interpretation:**   1. There is no local data on the incidence rate of prolonged neonatal jaundice. 2. Late diagnosis of biliary atresia remained a problem locally and internationally. 3. Clinical examination remained an important tool for the diagnosis of biliary atresia. 4. Stool colour chart registry has been successful in bringing down the age of diagnosis of biliary atresia. 5. By doing thyroid function test for prolonged neonatal jaundice babies, this might help in detecting babies with congenital hypothyroidism that were missed by the national screening programme. | | | | | |
