# Supplementary material for: Impact of a standardized protocol for the Management of Prolonged Neonatal Jaundice in a regional setting: an interventional quasi-experimental study
Source: BMC Pediatr. 2019 May 29;19:174. doi: 10.1186/s12887-019-1550-3 (PMC6540519; doi:10.1186/s12887-019-1550-3)
Supplement: Supplementary file 5 — Table S4: Comparison of American Association of Paediatrics and NICE guidelines in the management urinary tract infection (DOCX 21 kb) [file 12887_2019_1550_MOESM5_ESM.docx]

# Additional file 5: Table S4: Comparison of American Association of Pediatrics and NICE guidelines in the management urinary tract infection

| **Initial clinical questions:** | | Urinary tract infection remained a common problem in babies and as one of the causes for prolonged neonatal jaundice.   1. Is urine testing necessary during the initial assessment of prolonged neonatal jaundice? 2. If yes, what should be the urine sampling method? And which should we do, urine culture and urine microscopy OR urine microscopy alone? 3. What is the diagnostic criteria for UTI in young babies? | |
| --- | --- | --- | --- |
| **Institution** | | **American Association of Pediatrics 2011** [[39](#_ENREF_39)] | **NICE Clinical Guidelines 2007 [**[**18**](#_ENREF_18)**]** |
| **Guidelines** | | **Clinical Practice Guidelines for the Diagnosis and Management of the Initial Urinary Tract Infection in Febrile Infants and Children 2 to 24 Month** | **Urinary Tract Infection in Children: Diagnosis, Treatment and Long-Term Management** |
| **Area of Discussion** | |  |  |
| Diagnosis of UTI | Statement | To establish the diagnosis of UTI, clinicians should require both urine dipstick & microscopy test results that suggest infection (pyuria and/ or bacteriuria) AND the presence of at least 50000 colony-forming units (CFUs) per ml of a uropathogen cultured from a urine specimen obtained through catheterization or SPA (evidence quality C; recommendation) | For infants < 3 months, the most common symptoms and signs are fever, vomiting, lethargy and irritability; second are poor feeding and failure to thrive; while least common are abdominal pain, jaundice, haematuria and offensive urine. |
|  |  | Accurate diagnosis of UTI can prevent the spread of infection and renal scarring; avoiding over diagnosis of UTI can prevent overtreatment and unnecessary and expensive imaging. | Infants younger than 3 months with a possible UTI should be referred immediately to the care of a paediatric specialist. |
| Type of Urine Test | Statement | Urine dipstick & microscopy test cannot substitute for urine culture to document the presence of UTI but needs to be used in conjunction with culture. The key to distinguishing true UTI from asymptomatic bacteriuria is the presence of pyuria. | Indications for culture:   - diagnosis of acute pyelonephritis/upper urinary tract infection - high to intermediate risk of serious illness - under 3 years - a single positive result for leukocyte esterase or nitrite - recurrent UTI - infection that does not respond to treatment within 24–48 hours - Clinical symptoms and dipstick tests do not correlate. |
|  | Explanation | A nitrite test is not a sensitive marker for children. Negative nitrite test results have little value in ruling out UTI. The test is helpful when the result is positive, however, because it is highly specific.  The sensitivity of the leukocyte esterase test is 94% when it used in the context of clinically suspected UTI. The specificity of the leukocyte esterase test, averagely 72% (range:64%–92%), generally is not as good as the sensitivity, which reflects the non-specificity of pyuria in general  With numerous conditions other than UTI, including fever resulting from other conditions and after vigorous exercise, white blood cells may be found in the urine. Therefore, a finding of pyuria by no means confirms that an infection of the urinary tract is present.  More likely explanations for significant bacteriuria in culture in the absence of pyuria include contaminated specimens and asymptomatic bacteriuria | Test urine sample in babies and children:   - with symptoms and signs of UTI - with unexplained fever of 38°C or higher (test urine after 24 hours at the latest). - with an alternative site of infection but who remain unwell (consider urine test after 24 hours at the latest). |
| Urine Sampling Method | Statement | Reliable urine specimens for culture cannot be obtained without invasive methods. Invasive methods include urethral catheterization or suprapubic aspiration (SPA)  SPA has been considered the standard method for obtaining urine that is uncontaminated by perineal flora. Variable success rates for obtaining urine have been reported (23%–90%). When ultrasonographic guidance is used, success rates improve  Urine obtained through catheterization for culture has a sensitivity of 95% and a specificity of 99%, compared with that obtained through SPA  Urine dipstick & microscopy test can be performed on any specimen, including one collected from a bag applied to the perineum. | A clean catch urine sample is the recommended method for urine collection. If a clean catch urine sample is unobtainable: -  Other non-invasive methods such as urine collection pads should be used. It is important to follow the manufacturers’ instructions when using urine collection pads. Cotton wool balls, gauze and sanitary towels should not be used to collect urine in babies and children.  When it is not possible or practical to collect urine by non-invasive methods, catheter samples or SPA should be used.  Before SPA is attempted, ultrasound guidance should be used to demonstrate the presence of urine in the bladder. |
