# Supplementary material for: Impact of a standardized protocol for the Management of Prolonged Neonatal Jaundice in a regional setting: an interventional quasi-experimental study
Source: BMC Pediatr. 2019 May 29;19:174. doi: 10.1186/s12887-019-1550-3 (PMC6540519; doi:10.1186/s12887-019-1550-3)
Supplement: Supplementary file 6 — Table S5: Comparisons between other studies in the management of urinary tract infection in babies with jaundice. (DOCX 35 kb) [file 12887_2019_1550_MOESM6_ESM.docx]

# Additional file 6: Table S5: Comparisons between other studies in the management of urinary tract infection in babies with jaundice

| **No** | **Authors** | **Title** | **Study Type** | **Methodology** | **Results** | **Conclusion** | **Comments** |
| --- | --- | --- | --- | --- | --- | --- | --- |
| 1 | Garcia FJ,  *et al*  (United States) [[40](#_ENREF_40)] | Jaundice as an early diagnostic sign of UTI in infancy  *Pediatrics. 2002 May; 109(5): 846-51* | Prospective descriptive study | Population: Asymptomatic jaundiced infants younger than 8 weeks (n=160)  UTI (>10000 CFU/ml)  Urine sampling method: bladder catheterization | 12 out of 160 (7.5%) had UTI  Patients with onset of jaundice after 8 days old had higher incidence of UTI  Abnormal urine dipstick & microscopy test in 5/12 (42%) of UTI infants.  6 out of 11 (55%) of 11 infants had abnormal renal ultrasound | Recommend testing for UTI included in evaluation of asymptomatic, jaundice infants. | No control group  Did not subgroup the early and late onset |
| 2 | Fang SB,  *et al*  (Taiwan) [[41](#_ENREF_41)] | Urinary tract infections in young infants with prolonged neonatal jaundice.  *Acta Paediatrica Taiwanica. 46(6):356-60, 2005 Nov-Dec.* | Retrospective case-control | Sample: 50 infants 1 week to 3 months old with UTI and prolonged neonatal jaundice VS UTI only | Case group had lower incidence of high fever (12%), were less likely to have pyuria (29%), had fewer Escherichia coli but more Enterococcus species infections, and had more mixed infections (32%) than did the historical control. | High fever and pyuria are unreliable criteria for screening for UTI in young infants presenting with PNNJ. | Mixed infections → contamination? Not true UTI?  Retrospective study → measurement bias (urine sampling method?) |
| 3 | Ghaemi S,  *et al*  (Iran) [[42](#_ENREF_42)] | Late onset jaundice and UTI in neonates  *Indian J Pediatr. 2007 Feb; 74(2): 139-41* | Prospective descriptive study | Population: neonates with late onset jaundice admitted to 2 referral hospitals over 9 months. (n=400)  Urine sampling method: not mentioned | 23 out of 400 (5.8%) had UTI. The relation between type of feeding, circumcision and UTI was significant (*p*<0.05)  4 out of 23 (17%) of babies with UTI have urogenital abnormality | Evaluation of UTI should be considered as a screening test in all cases of neonatal late onset jaundice | Urine sampling method? |
| 4 | Satwik V, *et al*  (United Kingdom) [[43](#_ENREF_43)] | An audit of urine culture results in well infants attending a prolong jaundice clinic  *Arch Dis Child 2009; 94: 914.* | Retrospective study | Infants with PNNJ from 2002-2007. All have detailed history, exam & urine culture & sensitivity test  Infants with possible UTI (presence of white cells and/or growth> 10x10^6/^L) were grouped according to presence or absence of white cells in urine.  The possible UTI infants are recultured and if UTI were treated. | 14 out of 256 (5.5%) had UTI  On recultured, only 2/256 (0.8%) had UTI.  1 had white cells and found to have grade III reflux  The other had no white cells and found normal renal system. | Urine culture rarely positive in well infants.  Recommend that urine dipstick might prove to be more beneficial and cost-effective practice. | Urine sampling method? |
| 5 | Pashapour N, *et al*  (Iran) [[44](#_ENREF_44)] | UTI in term neonates with prolonged neonatal jaundice  *Urol J. 2007 Spring:4(2): 91-4* | Prospective study | Population: babies with > 2 weeks jaundice, asymptomatic. (n=100)  Urine samples: urine dipstick & microscopy test and urine culture (SPA). | 6 out of 100 (6%) had UTI. Out of 6, 1 had reflux, 2 had cortical defect in the kidney.  No significant difference in Sensitivity & Specificity in jaundice babies with or without UTI. | Urine culture should be a routine procedure in every babies with prolonged neonatal jaundice |  |
| 6 | Jafarzadeh M, *et al* [[45](#_ENREF_45)] | Should urine culture be considered in the hyperbilirubinaemia workup of neonate  (Journal Chinese Clinical Medicine 2009: 4(3): 136-138) | Prospective study | Population: Asymptomatic, jaundice neonates. (n=85)  Urine sampling method: SPA | 7 out of 85 (8.2%) had UTI.  No correlation between total and direct serum bilirubin level and positive urine culture. | More studies to answer this question |  |
| 7 | Eslami Z, *et al*  (Iran) [[46](#_ENREF_46)] | Investigation of Urinary Tract Infection in Neonates with Hyperbilirubinemia  *J Med Sci 2007; 7(5): 909-912* | Prospective case-control study | Population: 100 jaundiced and 100 non-jaundiced  History/ Physical examination was done. Babies unwell were excluded  Urine dipstick & microscopy test and Urine culture by bladder catheterization | 11 out of 100 jaundiced had UTI.  0 out of 100 non-jaundiced had UTI (stats significant)  3 out of 11 of positive urine growth had pyuria.  3 out of 11 of UTI cases had VUR. | UTI is one of the important factors in neonatal jaundice.  Necessary to do urine dipstick & microscopy test and urine culture in jaundiced babies. | Mean age in case group: 8.8 days  Mean age in control group: 14.7 days |
| 8 | Afzal N, et al  (Pakistan) [[47](#_ENREF_47)] | Urinary tract infection presenting as jaundice in neonates  *J Pak Med Assoc 2012; 62(7):* | Case studies | 5 cases that presented with idiopathic jaundice in the second week of life (at 12/10/7/12/13 days of life).  Urine samples via bladder catheterization. | All 5 (clinically well jaundiced babies) had unconjugated hyperbilirubinaemia and a diagnosis of UTI  4out of 5 ultrasound normal. | UTI should be considered as a cause of neonatal jaundice especially when indirect bilirubin peaks after one week of life. | More of getting urine samples in < 14 days jaundiced babies |
| 9 | Glissmeyer EW, et al  (Utah, United States) [[37](#_ENREF_37)] | Dipstick Screening for Urinary Tract Infection in Febrile Infants  Paediatrics 2014;133:e1121–e1127 | Retrospective | Population: Febrile infants aged 1-90 days from Intermountain Healthcare data warehouse  To identify febrile infants with urine dipstick, microscopy, and culture (2004- 2011)  UTI: >50 000 CFU per milliliter of a urinary pathogen.  Comparison of   1. urine dipstick alone 2. microscopy alone or 3. both tests combined (“combined urine dipstick & microscopy test”)   To identify UTI in infants aged 1 to 90 days.  Dipstick positive if either leukocyte esterase or nitrite was positive.  Microscopy positive if under high power field the technician observed either >10 white blood cells or any bacteria. | 13030 febrile infants identified.  6394 (49%) had all tests performed and were included. Of these, 770 (12%) had UTI.  Urine culture results were positive within 24 hours in 83% of UTIs.  Negative predictive value was >98% for all tests.  The combined urine dipstick & microscopy test NPV was 99.2% (95% confidence interval: 99.1%–99.3%) and was significantly greater than the dipstick negative predictive value of 98.7% (98.6%– 98.8%).  The urine dipstick PPV was significantly greater than combined urine dipstick & microscopy test (66.8% [66.2%–67.4%] vs 51.2% [50.6%– 51.8%]).  These data suggest 8 febrile infants would be predicted to have a false-positive combined urine dipstick & microscopy test for every 1 infant with UTI initially missed by dipstick screening.  Method sampling: catheterization | Urine dipstick testing compares favourably with both microscopy and combined urine dipstick & microscopy test in febrile infants aged 1 to 90 days.  The urine dipstick test may be an adequate stand-alone screening for UTI in febrile infants while awaiting urine culture results, with very high negative predictive value | First large study that had looked into the role of stand-alone urine dipstick & microscopy test in the screening of UTI in infants < 3 months old. |
| 10 | Schroeder AR, *et al*.  (United States) [[38](#_ENREF_38)] | Diagnostic Accuracy of the Urinalysis for Urinary Tract Infection in Infants <3 Months of Age  PEDIATRICS Volume 135, number 6, June 2015 | Retrospective | Population: 276 infants <3 months with bacteremic UTI from 11 hospital systems.  Sensitivity calculated on infants with at least a partial UA performed and had >50 000 colony-forming units per milliliter from the urine culture.  Specificity determined by a random sample of infants from the central study site with negative urine cultures. | 245 infants with bacteremic UTI and 115 infants with negative urine cultures.  Sensitivity of leukocyte esterase was 97.6% (95% confidence interval [CI] 94.5%–99.2%) and of pyuria (>3 white blood cells/high-power field) was 96% (95% CI 92.5%–98.1%).  1 infant with bacteremic UTI (Group B Streptococcus) and a complete urine dipstick & microscopy test had an entirely negative result.  In infants with negative urine cultures, leukocyte esterase specificity was 93.9% (95% CI 87.9 – 97.5) and of pyuria was 91.3% (84.6%–95.6%).  Method sampling: almost all catheterization | In young infants with bacteremic UTI, urine dipstick & microscopy test sensitivity is higher than previous reports.  In infants <3 months with bacteremic urinary tract infection, a condition that represents true infection, the urine dipstick & microscopy test sensitivity is higher than previously reported for urinary tract infection in general, suggesting that the urine dipstick & microscopy test is reliable even in young infants | Again, there is a role of urine dipstick & microscopy test in the screening for UTI in young infants. However, it is unsure if these infants were symptomatic or not. |
| **Summary:**   1. Mostly are small descriptive studies, with varied urine sampling and diagnostic criteria for UTI 2. However, for those studies with UTI diagnosed with bladder catheterization/ SPA with or without urine dipstick & microscopy test, the rate of UTI in well, jaundiced babies ranged from 5-8%. The rate of UTI in non-jaundiced babies remained unconfirmed. 3. Most studies agreed that UTI is one of the causes of prolonged neonatal jaundice, even in well babies. Some recommended both urine culture and urine dipstick & microscopy test to be done for this matter, while a few believed that urine dipstick & microscopy test is equally beneficial and cost-effective. | | | | | | | |
| **Interpretation:**   1. To confirm a UTI in babies, both positive culture and urine dipstick & microscopy test are needed. 2. Clean-catch method remained the safer way for culture but less specific and time-consuming if compared to bladder catheterization or SPA (invasive method). 3. UTI rates in well jaundiced babies had been quoted as 5-21%. Whether it is good practice to do urine culture (rendering well babies to repeated clean-catch urine test to invasive SPA) remain unanswered. 4. There is a role of urine dipstick & microscopy test, which can be done even with urine bag, in the screening of UTI in well, jaundiced babies. | | | | | | | |
